# Autonomic Dysfunction in Patients with *Bartonella henselae* IgM Seroreactivity: A Cross-Sectional Study

**DOI:** 10.3390/pathogens15070762

**Published:** 2026-07-20

**Authors:** Branislav Milovanović, Nikola Marković, Elizabeta Ristanović, Nikoleta Đorđevski, Sonja Atanasievska Kujović, Sulin Bulatović, Vasko Žugić, Maša Petrović, Milovan Bojić

**Affiliations:** 1 Institute for Cardiovascular Diseases “Dedinje”, 11000 Belgrade, Serbia; 2 School of Medicine, University of Belgrade, 11000 Belgrade, Serbia; 3 Institute for Microbiology, Military Medical Academy, 11000 Belgrade, Serbia; 4 Faculty of Medicine, University of Banja Luka, 78000 Banja Luka, Bosnia and Herzegovina

**Keywords:** *Bartonella henselae*, autonomic nervous system, heart rate variability, orthostatic intolerance, ambulatory blood pressure monitoring

## Abstract

**Background**: *Bartonella henselae* infection has been associated with a broad spectrum of neurological and autonomic manifestations, although its impact on autonomic nervous system function remains insufficiently characterized, and the aim of this study was to evaluate autonomic function in patients with polymorphic symptoms and *Bartonella henselae* IgM seroreactivity. **Methods**: In this cross-sectional study, 75 patients were compared with 75 age- and sex-matched healthy controls, and all participants underwent cardiovascular autonomic reflex testing, short-term (5 min) and long-term (24 h) heart rate variability analysis, and 24 h ambulatory blood pressure monitoring, while head-up tilt testing was performed in the Bartonella group. **Results**: Abnormal autonomic reflex tests were significantly more frequent in the *Bartonella IgM-seroreactive group*, particularly those reflecting parasympathetic function, while heart rate variability analysis demonstrated reduced high-frequency components and lower long-term variability indices, and head-up tilt testing revealed heterogeneous hemodynamic responses including orthostatic hypotension and pronounced blood pressure variability, with ambulatory monitoring additionally showing higher nighttime blood pressure values and reduced nocturnal dipping. **Conclusions**: These findings indicate a consistent pattern of autonomic imbalance characterized predominantly by parasympathetic impairment and altered blood pressure regulation in patients with *Bartonella henselae* IgM seroreactivity, although further studies are required to clarify underlying mechanisms and clinical implications.

## 1. Introduction

*Bartonella* is a fastidious, facultative intracellular, Gram-negative bacterium responsible for a wide spectrum of human diseases [1]. It is estimated that more than 40 subspecies exist, of which at least 16 have been identified as potential human pathogens, with the three most common being *Bartonella bacilliformis*, *Bartonella quintana*, and *Bartonella henselae* [1,2].

Among these, *Bartonella henselae* has received the greatest attention, with cat-scratch disease (CSD) representing its primary clinical manifestation. The incidence of CSD is approximately five cases per 100,000 population, with around 5% require hospitalization in the United States [1,2]. However, *Bartonella henselae* infection may present through a broad spectrum of atypical manifestations, including neurological, ocular, hepatosplenic, and musculoskeletal involvement, suggesting that the clinical burden of Bartonella-associated disease may extend beyond cases presenting as classical CSD [1,2]. However, clinical presentation is highly variable and largely dependent on the interaction between the pathogen and the host immune response [1,2]. *Bartonella* species are capable of invading erythrocytes, vascular endothelial cells, and other cell types, including monocytes, macrophages, and dendritic cells. This intracellular persistence may result in low-grade and potentially intermittent bacteremia in both cats and humans [1,2,3,4,5,6,7,8]. In this context, in addition to the typical clinical presentation, 10–25% of patients develop atypical manifestations (extranodal involvement affecting various organs), ranging from Parinaud’s oculoglandular syndrome and hepatosplenic involvement to a broad spectrum of neurological manifestations (including encephalitis, seizures, myelitis, and peripheral neuropathies), as well as endocarditis [1,2,3,4].

However, following the COVID-19 pandemic, particularly in the era of post-COVID conditions, with the recognition of a growing number of patients presenting with neurocognitive symptoms, the focus of medical research has increasingly shifted toward the infectious etiology of various neurological disorders [9,10,11]. Special emphasis has been placed on the impact of post-COVID conditions on the function of the autonomic nervous system (ANS), particularly the development of dysautonomia. The ANS represents the principal regulatory network responsible for involuntary control of non-motor organ systems, and numerous studies have investigated autonomic dysfunction, its prevalence, pathophysiology, and underlying mechanisms, particularly in patients with COVID-19 and post-COVID syndrome [12,13,14,15].

Beyond SARS-CoV-2, several other microorganisms have been associated with ANS dysfunction and the development of dysautonomia, including its major clinical manifestations such as syncope, postural orthostatic tachycardia syndrome (POTS), and orthostatic hypotension (OH). These include bacteria such as *Borrelia burgdorferi*, *Coxiella burnetii*, *Brucella* spp., and *Mycoplasma pneumoniae*, as well as viruses including herpes simplex virus (HSV), Epstein–Barr virus (EBV), cytomegalovirus (CMV), and varicella-zoster virus (VZV), each associated with distinct clinical profiles and phenotypes of dysautonomia [16,17,18,19,20,21,22,23,24,25,26,27,28,29,30,31].

In contrast, the impact of *Bartonella* on the function and dysfunction of the ANS is poorly studied. Current evidence is limited, with only a small number of case reports describing complex regional pain syndrome and autonomic disturbances in acute transverse myelitis [3]. It has been proposed that, in systemic *Bartonella* infection, damage to the nervous system may occur both as a direct consequence of the bacterium—through invasion of vascular endothelium and other cells of the microvasculature—and as a result of the host immune response, leading to disruption of the blood–brain barrier, local inflammation, ischemia, and subsequent neural injury [3,5].

The aim of this study was to investigate ANS function in patients presenting with a broad spectrum of polymorphic symptoms, including fatigue, cognitive dysfunction, myalgia, exercise intolerance, and myalgic encephalomyelitis/chronic fatigue syndrome (ME/CFS)-like features, as well as clinical manifestations suggestive of dysautonomia such as syncope, OH or intolerance, POTS palpitations, and blood pressure variability, who demonstrated *Bartonella henselae* IgM seroreactivity, in the absence of a previously recognized or typical clinical presentation of CSD.

## 2. Materials and Methods

### 2.1. Patient Enrollment

This cross-sectional study included adult participants (≥18 years) evaluated at the Neurocardiology Laboratory of the Cardiology Clinic, Institute for Cardiovascular Diseases “Dedinje”. Patients were consecutively referred for assessment due to a broad spectrum of polymorphic symptoms (e.g., fatigue, cognitive impairment, arthralgia, myalgia, sleep disturbances, and low-grade fever) and/or clinical features suggestive of autonomic dysfunction, including syncope, orthostatic hypotension (OH), or postural orthostatic tachycardia syndrome (POTS). Clinical diagnoses were established in accordance with current guidelines [32,33,34,35,36]. Between October 2022 and December 2025, a total of 1319 consecutively referred patients underwent serological testing as part of the diagnostic evaluation.

All participants underwent standardized autonomic nervous system (ANS) testing at the initial visit. As part of the same diagnostic pathway, patients were subsequently referred to the Military Medical Academy (Belgrade, Serbia), a national reference institution for infectious disease diagnostics, which were typically performed within 2–5 days.

Inclusion in the *Bartonella* IgM-seroreactive group required the presence of relevant clinical symptoms together with serological evidence of infection, defined as positive IgM antibodies against *Bartonella henselae*. Serological testing was performed using a commercial indirect immunofluorescence assay (IFA) (EUROIMMUN, Lübeck, Germany), utilizing *Bartonella henselae* grown in infected cells as the antigen substrate. Both IgM and IgG antibodies were assessed by the reference laboratory; however, only IgM seroreactivity was used as the inclusion criterion for the present study. Results were expressed as titers, and a cutoff value of ≥1:100 was considered positive in accordance with the manufacturer’s instructions. Based on previous studies, the specificity of Bartonella IgM antibodies has been reported to reach up to approximately 93%, while sensitivity ranges between 42% and 53% [37,38]. Similar findings were reported in a subsequent validation study of a EUROIMMUN indirect immunofluorescence assay utilizing cell-grown *Bartonella henselae* antigen and the same positivity cutoff (1:100), performed in patients with PCR-confirmed cat-scratch disease and PCR-negative controls with alternative diagnoses, which demonstrated IgM sensitivities of 50–62% and specificities of 87–96% [39]. IgM antibodies are typically detectable in the early phase of infection, with levels declining within approximately three months in the majority of patients, and are considered most informative within the first six weeks following symptom onset [37,38].

Among the 1319 tested patients, 75 (5.7%) met these criteria and were included in the Bartonella IgM-seroreactive group. The control group consisted of healthy volunteers recruited during the same period, from whom 75 age- and sex-matched individuals were selected for the present analysis. Healthy controls had no history of syncope, ME/CFS, post-COVID syndrome, autonomic disorders, or other chronic medical conditions known to affect autonomic nervous system function.

Given the clinical heterogeneity of the Bartonella IgM-seroreactive cohort, additional subgroup analyses were planned according to the presence or absence of previous syncope and ME/CFS which represented the two most prevalent clinical entities within the study population.

Exclusion criteria for the *Bartonella IgM-seroreactive group* included: (1) the presence of chronic medical conditions (e.g., neurological, endocrine, cardiological, rheumatological, or autoimmune disorders) that could account for the reported symptoms; (2) neurodegenerative diseases associated with primary autonomic failure, including pure autonomic failure, multiple system atrophy, and Parkinson’s disease; and (3) potential serological cross-reactivity of IgM antibodies with antigens from other infectious agents. Particular attention was given to excluding infections known to exhibit serological cross-reactivity with *Bartonella*, including *Borrelia burgdorferi*, *Coxiella burnetii*, *Chlamydia* spp., *Rickettsia* spp., and *Epstein-Barr virus*. All patients underwent comprehensive diagnostic evaluation, often involving repeated assessments by neurologists, rheumatologists, endocrinologists, and other specialists, in order to rigorously exclude alternative etiologies.

### 2.2. Ethical Approval

This study was approved by the Ethics Committee of the Institute for Cardiovascular Diseases “Dedinje” (approval No. 6472, dated 11 December 2024, and approval No. 1350, dated 13 March 2026) and conducted in accordance with the Declaration of Helsinki. It was supported by grant 451-03-68/2020-14/200156 from the Ministry of Education, Science and Technological Development of the Republic of Serbia, and by the COVANSA grant from the Science Fund of the Republic of Serbia.

### 2.3. Study Protocol

All participants underwent a comprehensive battery of diagnostic assessments for ANS function, including Ewing’s cardiovascular autonomic reflex tests (CARTs), short-term (5 min) ECG recording with short-term heart rate variability (HRV) analysis in resting, supine position, and 24 h Holter ECG monitoring with long-term HRV analysis, followed by 24 h ambulatory blood pressure monitoring in both groups. The head-up tilt test (HUTT) was performed exclusively in the Bartonella group. At the time of autonomic testing, participants were not using medications known to significantly affect ANS function (e.g., beta-blockers, calcium channel blockers, antihypertensive agents, anticholinergics, or sympathomimetics, etc.).

#### 2.3.1. Cardiovascular Autonomic Reflex Tests

Cardiovascular autonomic reflex testing comprised five standardized procedures designed to evaluate both sympathetic and parasympathetic components of the ANS. Sympathetic function was assessed using the handgrip test (HGT) and orthostatic blood pressure response (OH), while parasympathetic function was evaluated through the Valsalva maneuver (VM), heart rate response to deep breathing (HRB), and heart rate response to standing (HRS) [40]. According to Ewing’s classification, test outcomes are traditionally categorized as normal, borderline, or abnormal [40]. In line with more recent recommendations, borderline findings were interpreted as normal to minimize the impact of potential methodological variability [41]. For quantitative analysis, each test was scored as follows: normal = 0, borderline = 1, and abnormal = 2, yielding a total possible score ranging from 0 to 10 [39]. Sympathetic dysfunction was defined by the presence of at least one abnormal sympathetic test [39]. Parasympathetic involvement was graded based on the number of abnormal results, with a single abnormal test indicating early dysfunction and two or more abnormal tests considered indicative of established parasympathetic impairment [40].

#### 2.3.2. Head-Up Tilt Test

The HUTT was performed in accordance with the Westminster protocol [42]. Following a stabilization period of 10 min in the supine position, participants were tilted to an angle of 70° and maintained in this position for a maximum of 30 min. Throughout the procedure, continuous non-invasive blood pressure monitoring and 12-lead electrocardiographic recording were carried out. The test was considered positive in the event of syncope or the development of pronounced presyncopal symptoms. In addition to positive or negative outcomes, several hemodynamic responses were recorded during the test. Extreme variation in systolic blood pressure was defined as a sustained fluctuation greater than 20 mmHg between the maximum and minimum values in either the supine or passive phases, while small variation was defined as a fluctuation between 10 and 20 mmHg. A hypertensive reaction was defined as a sustained blood pressure exceeding 140/90 mmHg during the passive phase, whereas an extreme hypertensive reaction was defined as a sustained blood pressure exceeding 170/120 mmHg. POTS was defined as a rapid increase in heart rate of more than 30 beats per minute or a heart rate exceeding 120 beats per minute within ten minutes of assuming the upright position in the absence of orthostatic hypotension but with accompanying symptoms of orthostatic intolerance [34,35]. OH was defined as a sustained decrease in systolic blood pressure greater than 20 mmHg, a decrease in diastolic pressure greater than 10 mmHg, or an absolute systolic pressure lower than 90 mmHg [33].

#### 2.3.3. Short-Term ECG Recording and HRV Analysis

Short-term ECG recording was performed using a standard 12-lead electrocardiogram in a supine resting position over a 5 min period. Data acquisition and analysis were conducted using a commercially available software package (DM Systems, Beijing, China). The following parameters were obtained: heart rate; SDNN (standard deviation of normal-to-normal RR intervals, reflecting overall HRV); RMSSD (root mean square of successive differences between adjacent RR intervals, reflecting parasympathetic activity); pNN50 (percentage of consecutive RR intervals differing by more than 50 ms, also reflecting parasympathetic modulation); total power (TP); very low frequency (VLF); low frequency (LF); high frequency (HF); and the LF/HF ratio. Signal processing and HRV analysis were performed in accordance with established international guidelines [43,44]. Participants were instructed to remain relaxed, avoid movement and speaking, and maintain spontaneous, steady breathing in order to minimize the influence of respiratory variability on HRV measurements.

#### 2.3.4. 24 h Holter ECG Monitoring

A three-lead 24 h Holter ECG recording was performed using a commercially available device and analyzed by an experienced cardiologist with dedicated software (DM Systems, Beijing, China). In addition to mean heart rate, long-term HRV parameters were assessed, including time-and frequency-domain indices as described for short-term analysis, with the addition of ultra-low frequency (ULF).

#### 2.3.5. 24 h Ambulatory Blood Pressure Monitoring

Ambulatory blood pressure monitoring was performed using an oscillometric device over a 24 h period. Blood pressure measurements were obtained at 30 min intervals during the daytime (8 AM–10 PM) and at 60 min intervals during the nighttime (10 PM–8 AM). The analyzed parameters included mean systolic blood pressure (SBP) and diastolic blood pressure (DBP) values for the entire 24 h period, as well as separately for daytime and nighttime. In addition, the nocturnal blood pressure decline (dipping) was calculated and expressed as a percentage.

### 2.4. Statistical Analysis

Data are presented as mean ± standard deviation (SD), median (Mdn) with interquartile range (IQR, 25–75%), or counts (percentage), depending on data type. Normality of continuous variables was assessed using the Smirnov test and by visual inspection of histograms and Q–Q plots. Group comparisons were performed using parametric tests (independent samples *t* test) or nonparametric tests (Chi Square test, Fisher’s exact test, and Mann–Whitney U test) for categorical or non-normally distributed continuous data. Statistical analyses were conducted using SPSS version 26.0. A two-sided *p* value < 0.05 was considered statistically significant.

A priori sensitivity analysis was performed using G*Power software (version 3.1.9.4) to estimate the detectable effect size based on the available sample. Assuming a two-tailed independent samples *t*-test, an alpha level of 0.05, and a statistical power of 80%, with 75 participants in each group, the calculated effect size (Cohen’s d) was 0.46, indicating the ability to detect a moderate effect.

## 3. Results

In Table 1, the demographic characteristics and comorbidities of the study population are presented. There were no statistically significant differences between the *Bartonella* and control groups in terms of age and sex distribution. The most common comorbidities in the *Bartonella* group were syncope and ME/CFS, while approximately 7% of patients had a diagnosis of post-COVID syndrome. In addition, around 20% of patients had a diagnosis of hypertension.

In Table 2, a list of the most frequent symptoms in the *Bartonella IgM-seroreactive group* are shown. The most represented one was malaise in about 80% of patients, followed by chronic fatigue and cognitive problems. Fever and night sweats were present in about 25–40% of patients. Furthermore, gastrointestinal and genitourinary symptoms were also frequently observed, including diarrhea and postprandial syndromes (both 33.3%), urinary symptoms and abdominal pain/cramping (both 25.3%), and constipation (17.3%).

**Figure 1:**
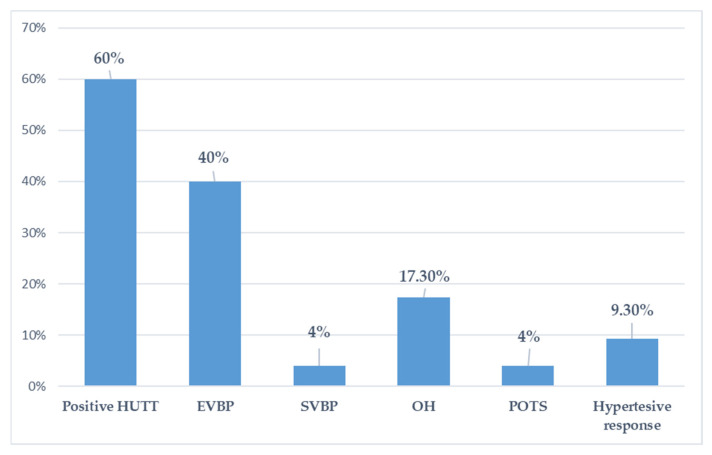
Responses during head-up tilt test in the *Bartonella IgM-seroreactive group*. HUTT—head-up tilt test; EVBP—extreme variation in blood pressure; SVBP—small variations in blood pressure; OH—orthostatic hypotension; POTS—postural orthostatic tachycardia syndrome.

In Figure 1, responses during HUTT in the *Bartonella IgM-seroreactive group* are shown. Positive HUTT was present in 60% of patients and 40% of patients had extreme variations in blood pressure (EVBP) during the test. Orthostatic hypotension was presented in about 17% of patients, while frequency of POTS was present in only about 4%.

**Figure 2:**
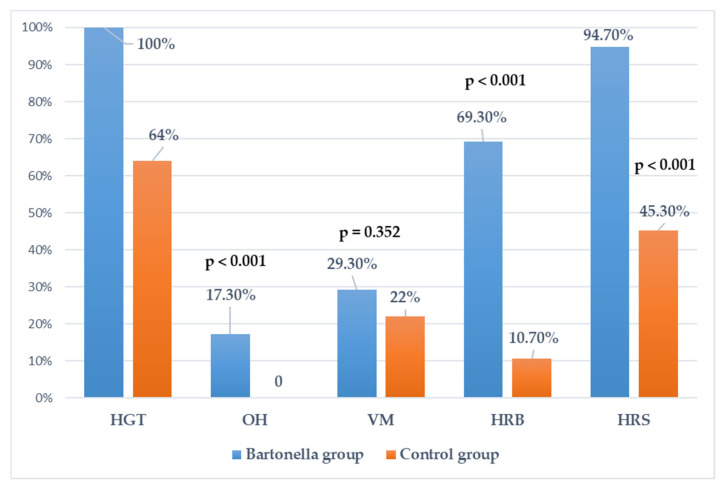
Abnormal cardiovascular autonomic reflex tests (CARTs) between groups in the study population: HGT—handgrip test; OH—orthostatic hypotension; VM—Valsalva maneuver; HRB—heart rate response to deep breathing; HRS—heart rate response to standing. *p* value responds to Pearson Chi Square test.

Figure 2 shows abnormal CART test results in the study population. All tests were significantly more frequently abnormal in the *Bartonella IgM-seroreactive group* compared to the control group, except for the Valsalva maneuver.

In Table 3, values of the short-term HRV parameters in the study population are shown. Most parameters showed comparable values between the two groups, without reaching statistical significance. However, HF values were significantly lower in the Bartonella group compared to the control group, whereas the LF/HF ratio was significantly higher (*p* < 0.05 for both parameters).

Table 4 presents the values of long-term HRV parameters in the study population. Although most parameters were lower in the Bartonella group, only LF, HF, and ULF were significantly reduced. In both groups, median LF/HF values were >2.

Table 5 presents the values of ambulatory blood pressure parameters in the study population. Most parameters were higher in the *Bartonella* group, with nighttime systolic and diastolic blood pressure values being significantly elevated. In contrast, the percentage of nocturnal dipping was lower in the *Bartonella* group.

Additional subgroup analyses according to the presence or absence of previous syncope are presented in Tables S1–S5. No significant differences were observed between groups with respect to age, sex distribution, cardiovascular autonomic reflex test results, or hemodynamic responses during head-up tilt testing (Tables S1 and S2). Similarly, short-term HRV parameters did not differ significantly between groups, with the exception of a lower LF/HF ratio in patients with previous syncope (Table S3). In contrast, long-term HRV analysis demonstrated significantly higher values of SDNN, RMSSD, pNN50, and ULF in patients with previous syncope (Table S4). Furthermore, ambulatory blood pressure monitoring revealed significantly lower 24 h, daytime, and nighttime diastolic blood pressure values in the syncope subgroup, whereas systolic blood pressure parameters and nocturnal dipping did not differ significantly between groups (Table S5).

Analyses according to the presence or absence of ME/CFS are presented in Tables S6–S10. No significant differences were observed between groups with respect to age, sex distribution, cardiovascular autonomic reflex test results, hemodynamic responses during head-up tilt testing, or ambulatory blood pressure monitoring parameters (Tables S6, S7 and S10). Similarly, long-term HRV parameters did not differ significantly between participants with and without ME/CFS (Table S9). In contrast, short-term HRV analysis demonstrated significantly lower values for rMSSD, pNN50, and HF power, together with a significantly higher LF/HF ratio in participants with ME/CFS. (Table S8).

## 4. Discussion

The findings of this study demonstrate a distinct pattern of autonomic abnormalities in patients presenting with polymorphic symptoms and *Bartonella henselae* IgM seroreactivity. The observed pattern was predominantly characterized by parasympathetic impairment, as evidenced by both short- and long-term heart rate variability analyses, as well as cardiovascular autonomic reflex testing. However, a subset of patients also demonstrated findings suggestive of sympathetic involvement, most notably orthostatic hypotension, as well as heterogeneous hemodynamic responses during head-up tilt testing and ambulatory blood pressure monitoring.

As shown in Table 1, the study population was predominantly female, with a mean age of approximately 45 years. Previous studies have reported involvement of both sexes, with some indicating male and others female predominance [6,45,46]. The observed female predominance in our cohort does not necessarily reflect a true sex-specific susceptibility to *Bartonella henselae IgM seroreactivity*, but may rather be influenced by the demographic characteristics of associated conditions, such as syncope and ME/CFS (Table 1), which are known to exhibit female predominance [47,48]. With regard to comorbidities, a history of syncope was present in approximately 42% of patients. The prevalence of syncope, as one of the key manifestations of dysautonomia, among individuals with evidence of *Bartonella* exposure or seroreactivity remains poorly characterized. Previous studies have reported the presence of *Bartonella*-specific IgG antibodies in 1.5% to 6% of patients with syncope, both with and without OH [20]. With respect to post-COVID syndrome, case reports have described the occurrence of complications related to CSD in patients with acute COVID-19, highlighting the complexity of managing overlapping infectious conditions in the context of a global pandemic [49]. In terms of the second-most common comorbidity in our cohort, namely ME/CFS, it is well established that in approximately two-thirds of patients, symptoms are preceded by an infectious trigger. Breitschwerdt et al. reported evidence of *Bartonella* and/or *Babesia* infection in up to 46% of patients with chronic fatigue [8,50]. The findings of the present study, together with previous reports, support the need for further investigation of a possible association between *Bartonella* spp. and ME/CFS; however, the available evidence remains insufficient to infer a causal relationship.

It should also be noted that precise data on the prevalence of *Bartonella* infection in the region where this study was conducted are not fully available. However, previous studies have demonstrated the presence of *Bartonella* in up to 57% of cats in this area, along with reports of ocular manifestations in the pediatric population [51,52]. Furthermore, epidemiological studies from neighboring Greece have reported *Bartonella* seroprevalence rates ranging from 15.9% to 19.8% in healthy populations, based on combined serological assessment of IgG and IgM antibodies against *Bartonella henselae* and *Bartonella quintana* [52]. Although these data are not directly comparable to the *Bartonella henselae* IgM-seroreactive population investigated in the present study, they suggest that exposure to *Bartonella* spp. may not be uncommon in this part of southeastern Europe [53].

A detailed overview of symptoms in this cohort is presented in Table 2. Malaise was the most frequently reported symptom, which is consistent with previous reports indicating its presence in approximately 25–50% of patients with acute infection [1,2]. Neurocognitive symptoms were also commonly observed (50–66%; Table 2), in line with the relatively frequent neurological involvement described in *Bartonella henselae* infection [1,2,3]. These findings are consistent with previous reports suggesting that neurological manifestations associated with *Bartonella* spp. may involve vascular, inflammatory, and immune-mediated mechanisms affecting the nervous system [3,5]. Previous histopathological studies have demonstrated granulomatous meningoencephalitis with prominent perivascular lymphocytic infiltrates, supporting the presence of inflammation localized around the microvasculature [3]. On the other hand, fever was present in approximately 25% of patients (Table 2). Fever is a common feature of acute infections and, in certain cases, may precede lymphadenopathy in classical cat-scratch disease [1,3]. It should be noted that the autonomic nervous system plays an important role in both thermoregulation and immune function [54,55]. In this context, parasympathetic activity—shown to be impaired in our patients—has a well-established anti-inflammatory role [54,55]. Gastrointestinal and genitourinary symptoms were also relatively common in our cohort, including diarrhea, postprandial syndromes, urinary symptoms, abdominal pain/cramping, and constipation (Table 2). Such symptoms have been described in a variety of conditions, including those involving autonomic dysfunction, and further illustrate the heterogeneous clinical presentation of the studied population. Taken together, these findings highlight the coexistence of autonomic abnormalities and a broad spectrum of systemic and neurological symptoms in this patient population. However, the relationship between these manifestations remains uncertain and requires further investigation. Furthermore, the heterogeneous and often prolonged duration of symptoms in this cohort precludes conclusions regarding the temporal relationship between symptom development and autonomic abnormalities.

As shown in Figure 1, a variety of hemodynamic responses during the HUTT were observed in the study cohort. A positive HUTT was present in 60% of patients, which may be consistent with the previously reported occurrence of syncope in this population (Table 1). Similar proportions have been reported in association with other infectious agents, such as *Coxiella burnetii* and *Borrelia burgdorferi*, with rates ranging from 50% to 55%, suggesting the presence of a certain degree of orthostatic intolerance across these groups [19,21]. The most frequently observed hemodynamic pattern was extreme blood pressure variability (EBPV), defined as a fluctuation amplitude greater than 20 mmHg (difference between maximal and minimal values), which represents another similarity with *Coxiella burnetii* infection [21]. Such variability may indicate altered autonomic regulation and impaired short-term blood pressure control, potentially involving baroreflex mechanisms [56,57]. Additionally, the presence of blood pressure variability during HUTT has been associated with a positive predictive value of approximately 80% for vasovagal syncope [58]. However, related to this study, these findings should be interpreted cautiously, as HUTT was not performed in the control group and direct comparisons could therefore not be made. Interestingly, approximately 4% of patients developed POTS during the HUTT, one of the main clinical manifestations of dysautonomia and orthostatic intolerance [34,35,59]. Among the observed hemodynamic patterns, POTS has been most consistently associated with infectious triggers, including SARS-CoV-2, *Borrelia burgdorferi*, *Mycoplasma pneumoniae*, and *Coxiella burnetii*, with reported prevalence ranging from 4% to 7% [21,34,35,59,60].

As shown in Figure 2, the HGT and OH were significantly more frequently abnormal in the *Bartonella* group. The HGT was abnormal in all patients. Although HGT is still included in autonomic testing batteries, it has been subject to considerable criticism, mainly due to the fact that many patients may inadvertently perform a Valsalva maneuver during the test, thereby compromising results, and because the outcomes are influenced by baseline arterial blood pressure levels [61,62,63]. Additionally, there have been suggestions that this test should be excluded from standard testing batteries [64]. In contrast, OH was present in approximately 17% of patients. OH represents a hallmark of sympathetic dysfunction, including impairment of baroreflex function, and has been associated with an increased risk of cardiovascular morbidity [65,66,67,68]. Previous studies have reported the presence of OH in approximately 50% of patients with Lyme disease and in about 13% of patients with *Coxiella burnetii* infection [16,21]. Both *Coxiella burnetii* and *Bartonella henselae* are intracellular bacteria, a feature that may contribute to their persistence and complex clinical presentation. Furthermore, Breitschwerdt et al. reported that up to 46% of patients with *Bartonella* spp. bacteremia had a prior diagnosis of Lyme disease, while Eskow et al. demonstrated cases of concurrent central nervous system infection with *Borrelia* and *Bartonella henselae* [45,69]. However, Milovanović et al. reported that *Bartonella henselae* IgM seroreactivity was a negative predictor of OH, although this finding did not retain statistical significance in multivariable analysis [20]. In the same analysis, patients with positive *Bartonella henselae* IgG antibodies were more likely to belong to the group with isolated orthostatic hypotension compared to those with orthostatic syncope [20]. Previous studies have suggested that preserved cerebral autoregulation may reduce the likelihood of symptom development and syncope in patients with orthostatic hypotension [70]. Although speculative, this mechanism may potentially contribute to the lower occurrence of syncope despite the presence of OH observed in these individuals. However, a more detailed pathophysiological explanation is beyond the scope of the present study.

Additionally, as shown in Figure 2, abnormal HRB and HRS were significantly more frequent in patients with *Bartonella*. An abnormal HRS, similarly to OH, indicates impaired orthostatic adaptation in these patients. In contrast, HRB essentially reflects the ability to generate respiratory sinus arrhythmia, which is mediated by the atrial (Bainbridge) reflex [71,72]. Abnormal findings in this test may indicate dysfunction within the reflex pathway responsible for respiratory sinus arrhythmia, encompassing both peripheral and central components of autonomic cardiovascular regulation.

The HRB findings are partly consistent with the results of both short- and long-term HRV analyses (Table 3 and Table 4), where HF values were significantly lower in patients with *Bartonella*. HF represents one of the most important parameters for assessing parasympathetic function and reflects the capacity to generate respiratory sinus arrhythmia (the so-called “respiratory band”), which is mediated by the previously described reflex pathway [43,44]. However, it should be noted that short- and long-term HRV, although assessing similar parameters, are not interchangeable. In short-term recordings, parasympathetic (vagal) influence predominates, particularly under resting, supine conditions, whereas long-term HRV reflects a greater contribution of sympathetic activity, capturing the effects of various physiological stressors over a 24 h period [44]. Still, similar findings have been reported in Lyme disease, where Puri et al. demonstrated reduced respiratory modulation of cardiac vagal tone, suggesting possible involvement of brainstem structures [18]. Given the observed consistency within our cohort, reflected by both HRB abnormalities and reduced HF values, these findings support the presence of parasympathetic abnormalities involving pathways responsible for respiratory sinus arrhythmia. However, the underlying mechanisms and their relationship to *Bartonella henselae* IgM seroreactivity remain to be determined.

LF, which was also lower during 24 h monitoring in patients with *Bartonella*, does not have as straightforward an interpretation as HF (Table 4). Although it was previously considered a marker of sympathetic activity, this parameter is now regarded as reflecting both sympathetic and parasympathetic influences, as well as baroreceptor function, depending on recording conditions [44]. Lower values observed in the *Bartonella* IgM-seroreactive group may therefore further suggest impairment of autonomic nervous system function, involving both sympathetic and parasympathetic components, with an additional contribution of baroreceptor dysfunction. In addition to LF and HF, the *Bartonella* group also demonstrated reduced ULF values (Table 4). ULF is generally associated with very slow, long-term regulatory processes, including circadian modulation and broader neurohumoral mechanisms [43,44]. Together with the observed changes in LF and HF, reduced ULF values may point toward broader disturbances in autonomic and neurohumoral regulation. However, the clinical and biological significance of these findings remains uncertain and requires further study.

Finally, Table 5 shows higher nighttime systolic blood pressure values, with consequently reduced nocturnal dipping in the *Bartonella* group. Hypertension itself has a complex and not fully understood pathophysiology; however, in addition to renal and vascular factors, excessive activation of the sympathetic nervous system plays a major role in its development [73]. The sympathetic nervous system (SNS) contributes to individual variability in nocturnal blood pressure dipping, with increased sympathetic activity associated with a blunted or absent nighttime decline; moreover, enhanced SNS activation is likely one of the key mechanisms underlying the altered day–night blood pressure pattern [74,75]. In addition, elevated nighttime blood pressure values and a disrupted dipping pattern have been consistently associated with increased cardiovascular risk [76,77]. Taken together, these findings, in conjunction with the observed abnormalities on cardiovascular reflex testing and HRV analysis, are consistent with altered autonomic regulation of blood pressure and may help explain the elevated nighttime values and reduced nocturnal dipping observed in the Bartonella IgM-seroreactive group.

Additional subgroup analyses according to previous syncope (Tables S1–S5) revealed largely comparable autonomic findings across most cardiovascular reflex tests, HUTT responses, and short-term HRV parameters. The only significant difference in short-term HRV was a lower LF/HF ratio among patients with previous syncope. Given the ongoing debate regarding the physiological interpretation of LF/HF, the significance of this isolated finding remains uncertain [44]. In contrast, long-term HRV analysis demonstrated higher SDNN, RMSSD, and pNN50 values in the syncope subgroup, a pattern that may be consistent with enhanced vagal modulation previously described in certain forms of vasovagal syncope [78,79]. Furthermore, patients with previous syncope exhibited lower 24 h, daytime, and nighttime diastolic blood pressure values. Interestingly, this finding differs from previous studies of reflex syncope, which generally reported higher diastolic blood pressure values and lower pulse pressure as part of a compensatory hemodynamic profile [80,81]. One possible explanation is that the autonomic alterations observed in Bartonella IgM-seroreactive patients may modify the hemodynamic characteristics typically associated with reflex syncope. However, given the exploratory nature of this subgroup analysis and the absence of mechanistic data, the significance of these differences remains uncertain. Importantly, despite these findings, no significant differences were observed in cardiovascular autonomic reflex testing or orthostatic hemodynamic responses, suggesting that the overall pattern of autonomic abnormalities identified in the *Bartonella* IgM-seroreactive cohort was not confined to patients with a history of syncope.

As shown in the ME/CFS subgroup analysis, participants with ME/CFS demonstrated significantly lower short-term vagally mediated HRV indices, including rMSSD, pNN50, and HF together with a higher LF/HF ratio. These findings are consistent with previous studies demonstrating reduced short-term HRV parameters in patients with ME/CFS compared with healthy controls [15,82], supporting the presence of impaired parasympathetic modulation in this condition. In contrast, no significant differences were observed in long-term HRV parameters between Bartonella IgM-reactive participants with and without ME/CFS. This finding is not entirely consistent with previous studies reporting lower HRV values in patients with ME/CFS, including those by Boneva et al., Nelson et al., and more recently Walitt et al. [83,84,85]. However, short-term and long-term HRV reflect partially distinct aspects of autonomic regulation. Short-term recordings predominantly assess resting parasympathetic activity, whereas long-term recordings are additionally influenced by circadian rhythms, physical activity, behavioral factors, and sympathetic influences [44]. Importantly, despite differences in short-term HRV, no significant differences were observed between participants with and without ME/CFS regarding cardiovascular autonomic reflex test results, head-up tilt testing responses, ambulatory blood pressure monitoring findings, or long-term HRV parameters. Therefore, while ME/CFS may contribute to impaired resting parasympathetic modulation, it is unlikely to fully explain the broader autonomic phenotype observed in the Bartonella IgM-reactive cohort.

### Study Limitations

This study has several important limitations: (1) Sample size—Future studies should include a larger cohort of patients, with improved age- and sex-matching of control groups to enhance the robustness of the findings. Additionally, longitudinal designs are warranted to evaluate temporal changes and the potential impact of therapeutic interventions. (2) Inclusion criteria—The study population was defined on the basis of isolated *Bartonella henselae* IgM seroreactivity without molecular confirmation. Although IgM antibodies may suggest recent exposure, serological testing is characterized by imperfect sensitivity and specificity and may be influenced by cross-reactivity with other infectious agents. Therefore, the present findings should be interpreted as an association between *Bartonella henselae* IgM seroreactivity and autonomic abnormalities rather than evidence of a direct causal relationship. Future research should aim to apply more precise diagnostic algorithms, ideally incorporating molecular methods such as PCR from appropriate biological samples (e.g., lymph node tissue or other relevant substrates), in addition to serological testing. Furthermore, studies focusing on patients with a more typical clinical presentation, such as classical cat-scratch disease, would provide clearer insights into disease-specific mechanisms. (3) Clinical heterogeneity—The Bartonella IgM-seroreactive cohort included participants with diverse clinical presentations, including ME/CFS, previous syncope, post-COVID syndrome, and other manifestations potentially associated with autonomic dysfunction. Although additional subgroup analyses according to the presence or absence of ME/CFS and previous syncope demonstrated broadly comparable findings across cardiovascular autonomic reflex testing, head-up tilt testing, ambulatory blood pressure monitoring, and most HRV parameters, residual confounding related to clinical heterogeneity cannot be entirely excluded. Larger studies focusing on more homogeneous patient populations will be required to further clarify the contribution of individual clinical entities to the observed autonomic abnormalities. (4) Additional diagnostic modalities—Future investigations should consider more comprehensive assessment of autonomic nervous system function, including baroreflex sensitivity (BRS), baroreflex effectiveness index (BEI), evaluation of small fiber neuropathy, as well as advanced cardiac autonomic markers such as deceleration and acceleration capacity and heart rate turbulence.

## 5. Conclusions

*Bartonella henselae*, beyond its classical presentation as cat-scratch disease, has been associated with a broad range of neurological and autonomic manifestations. In this study, patients with polymorphic symptoms and *Bartonella henselae* IgM seroreactivity demonstrated a consistent pattern of autonomic abnormalities across multiple diagnostic modalities. The observed profile was predominantly characterized by abnormalities in cardiovascular autonomic reflex tests and heart rate variability parameters, accompanied by heterogeneous hemodynamic responses during head-up tilt testing. Furthermore, ambulatory blood pressure monitoring revealed elevated nighttime blood pressure values and reduced nocturnal dipping, suggesting altered circadian blood pressure regulation. Additional subgroup analyses according to previous syncope and ME/CFS status demonstrated broadly comparable autonomic findings across most assessments. Taken together, these findings support the presence of autonomic involvement in this patient population. However, given the use of isolated IgM seroreactivity as the inclusion criterion, the results should be interpreted as an association rather than evidence of a direct causal relationship. Further longitudinal and mechanistic studies are required to clarify the underlying biological pathways and clinical significance of these findings.

## Figures and Tables

**Table 1:** Demographic characteristics and comorbidities in the study population.

	*Bartonella IgM-Seroreactive Group* N = 75	Control Group N = 75	*p* Value
Female (n, %)	53 (70.7%)	56 (74.7%)	0.583 ^c^
Age (mean ± SD)	45.6 ± 13.8	41.4 ± 13.2	0.061 ^t^
**Comorbidities**			
HTA (n, %)	17 (22.7%)	0	N/A
DM (n, %)	0	0	N/A
Asthma (n, %)	1 (1.3%)	0	N/A
PCS (n, %)	5 (6.7%)	0	N/A
ME/CFS (n, %)	23 (30.7%)	0	N/A
Syncope (n, %)	32 (42.7%)	0	N/A

SD—standard deviation; HTA—hypertension; DM—diabetes mellitus; PCS—post-COVID syndrome; ME/CFS—myalgic encephalomyelitis/chronic fatigue syndrome; N/A—not applicable; ^c^—Pearson Chi Square; ^t^—independent samples *t* test.

**Table 2:** List of most frequent symptoms in *Bartonella* group.

	*Bartonella IgM-Seroreactive Group* N = 75
Fever (n, %)	19 (25.3%)
Malaise (n, %)	62 (82.7%)
Night sweats (n, %)	31 (41.3%)
Muscle/joint pain (n, %)	31 (41.3%)
Headache (n, %)	31 (41.3%)
Chronic fatigue (n, %)	50 (66.7%)
Visual disturbances (n, %)	6 (8%)
Confusion, difficulty thinking (n, %)	38 (50.7%)
Forgetfulness (n, %)	50 (66.7%)
Irritability (n, %)	31 (41.3%)
Urinary symptoms * (n, %)	19 (25%)
Abdominal pain/cramping (n, %)	19 (25%)
Diarrhea (n, %)	25 (33.3%)
Constipation (n, %)	13 (17.3%)
Postprandial syndromes ** (n, %)	25 (33.3%)

*—urinary incontinence, incomplete bladder emptying, urinary frequency; **—loss of appetite, early satiety, persistent fullness, vomiting, frequent nausea.

**Table 3:** Values of short-term HRV parameters of the study population.

	*Bartonella IgM-Seroreactive Group* N = 75	Control Group N = 75	*p* Value
HR (bpm) (mean ± SD)	76.1 ± 13.3	74.5 ± 12.6	0.524 ^t^
SDNN (ms) (Mdn(IQR))	55 (38–72.5)	54 (37–72)	0.707 ^m^
RMSSD (ms) (Mdn(IQR))	35 (23.5–72.8)	32 (24–57)	0.378 ^m^
PNN50 (%)(Mdn(IQR))	5.5 (2–17)	11 (2–25)	0.158 ^m^
TP (ms^2^) (Mdn(IQR))	1250.5 (560.6–2258.4)	1254.3 (929.9–2041.5)	0.388 ^m^
VLF (ms^2^) (Mdn(IQR))	635.2 (270.3–1297.8)	471.9 (325.7–745)	0.446 ^m^
LF (ms^2^) (Mdn(IQR))	295 (147.5–556)	363 (204.8–575.2)	0.300 ^m^
HF (ms^2^) (Mdn(IQR))	120.8 (53.1–274.5)	223.9 (123.5–517.5)	0.001 ^m^
LF/HF (Mdn(IQR))	2.4 (1.6–4.1)	1.3 (0.5–2.5)	0.001 ^m^

HR—heart rate; bpm—beats per minute; SDNN—standard deviation of normal-to-normal RR intervals; RMSSD—root mean square of successive RR interval differences; pNN50—percentage of successive RR intervals differing by >50 ms; TP—total power; VLF—very low frequency; LF—low frequency; HF—high frequency; LF/HF—low frequency to high frequency ratio; ms—milliseconds; ms^2^—square milliseconds; SD—standard deviation; Mdn—median; IQR—interquartile range; ^t^—independent samples *t* test; ^m^—Mann–Whitney U test.

**Table 4:** Values of long-term HRV parameters of the study population.

	*Bartonella IgM-Seroreactive Group* N = 75	Control Group N = 75	*p* Value
HR (bpm) (Mdn(IQR))	75 (69.5–81.8)	77 (70–83)	0.584 ^m^
SDNN (ms) (Mdn(IQR))	150 (122.5–225.5)	157 (137.5–187.5)	0.951 ^m^
RMSSD (ms) (Mdn(IQR))	33 (16–70)	35 (27–46)	0.592 ^m^
PNN50 (%) (Mdn(IQR))	8 (0–24.5)	12 (7–21)	0.274 ^m^
TP (ms^2^) (Mdn(IQR))	2905.3 (1355.9–5663.9)	3751.1 (3097.5–4804.3)	0.068 ^m^
VLF (ms^2^) (Mdn(IQR))	2000.2 (864.9–3723.9)	2436.5 (1847.1–3395.3)	0.160 ^m^
LF (ms^2^) (Mdn(IQR))	518 (290.2–974.2)	927.5 (781.8–1174.2)	0.007 ^m^
HF (ms^2^) (Mdn(IQR))	159 (46.8–394.9)	316.7 (182.2–488.5)	0.027 ^m^
ULF (ms^2^) (Mdn(IQR))	11 (5.9–36.9)	23.1 (13.7–30.6)	0.042 ^m^
LF/HF (Mdn(IQR))	2.9 (2.2–5.1)	27 (2.1–3.8)	0.498 ^m^

HR—heart rate; bpm—beats per minute; SDNN—standard deviation of normal-to-normal RR intervals; RMSSD—root mean square of successive RR interval differences; pNN50—percentage of successive RR intervals differing by >50 ms; TP—total power; VLF—very low frequency; LF—low frequency; HF—high frequency; LF/HF—low frequency to high frequency ratio; ms—milliseconds; ULF—ultra-low frequency; ms^2^—square milliseconds; Mdn—median; IQR—interquartile range; ^m^—Mann–Whitney U test.

**Table 5:** Values of ambulatory blood pressure parameters of the study population.

	*Bartonella IgM-Seroreactive Group* N = 75	Control Group N = 75	*p* Value
SBP, average (mmHg) (mean ± SD)	118.9 ± 8.2	115.3 ± 8.5	0.154 ^t^
SBP, day (mmHg) (mean ± SD)	120 ± 8.5	118.3 ± 9.1	0.528 ^t^
SBP, night ((mmHg) (mean ± SD)	114.5 ± 8.4	105 ± 10.4	0.003 ^t^
DBP, average, (mmHg) (mean ± SD)	75.2 ± 9	72.2 ± 6.7	0.163 ^t^
DBP, day (mmHg) (mean ± SD)	77.6 ± 8	74.1 ± 6.5	0.090 ^t^
DBP, night (mmHg) (mean ± SD)	70.1 ± 8.3	64.5 ± 7.1	0.013 ^t^
SBP dipping (%) (Mdn(IQR))	6 (0.9–8)	10.4 (6.4–16.9)	0.007 ^m^

SBP—systolic blood pressure; DBP—diastolic blood pressure; SD—standard deviation; Mdn—median; IQR—interquartile range (25–75%); ^t^—independent samples *t* test; ^m^—Mann–Whitney U test.

## Data Availability

The data are available upon reasonable request.

## Supplementary Materials

The following supporting information can be downloaded at: https://www.mdpi.com/article/10.3390/pathogens15070762/s1, Table S1: Comparison of demographic characteristics and head-up tilt test findings according to history of syncope within the *Bartonella* IgM-seroreactive group; Table S2: Comparison of cardiovascular autonomic reflex test results according to history of syncope within the *Bartonella* IgM-seroreactive group; Table S3: Comparison of short-term heart rate variability according to history of syncope within the *Bartonella* IgM-seroreactive group; Table S4: Comparison of long-term heart rate variability according to history of syncope within the *Bartonella* IgM-seroreactive group; Table S5: Comparison of ambulatory blood pressure monitoring parameters according to history of syncope within the *Bartonella* IgM-seroreactive group; Table S6: Comparison of demographic characteristics and autonomic findings between *Bartonella henselae* IgM-reactive participants with and without myalgic encephalomyelitis/chronic fatigue syndrome (ME/CFS); Table S7: Comparison of cardiovascular autonomic reflex test (CART) abnormalities between *Bartonella henselae* IgM-reactive participants with and without myalgic encephalomyelitis/chronic fatigue syndrome (ME/CFS); Table S8: Comparison of short-term heart rate variability parameters between *Bartonella henselae* IgM-reactive participants with and without myalgic encephalomyelitis/chronic fatigue syndrome (ME/CFS); Table S9: Comparison of 24-hour heart rate variability parameters between *Bartonella henselae* IgM-reactive participants with and without myalgic encephalomyelitis/chronic fatigue syndrome (ME/CFS); Table S10: Comparison of 24-hour ambulatory blood pressure monitoring parameters between *Bartonella henselae* IgM-reactive participants with and without myalgic encephalomyelitis/chronic fatigue syndrome (ME/CFS).
