# Supplementary material for: Autonomic Dysfunction in Patients with Bartonella henselae IgM Seroreactivity: A Cross-Sectional Study
Source: Pathogens. 2026 Jul 20;15(7):762. doi: 10.3390/pathogens15070762 (PMC13414667; doi:10.3390/pathogens15070762)
Supplement: Supplementary file 1 [file pathogens-15-00762-s001.zip › pathogens-4328311-supplementary.pdf]

Table S1. Comparison of demographic characteristics and head-up tilt test findings according to history of syncope within the Bartonella IgM-seroreactive group.

	No history of Syncope N = 43	Previous Syncope N = 32	p
**Demographic characteristcs**			
Age (yrs.) (mean ± SD)	44.9 ± 10.3	46.6 ± 17.6	.628[t]
Female (n,%)	34 (79.1%)	19 (59.4%)	.077[c]
**Responses during HUTT**			
Positive HUTT (n,%)	26 (60.5%)	19 (59.4%)	.924[c]
EBPV (n,%)	15 (34.9%)	15 (46.9%)	.294[c]
SBPV (n,%)	3 (7%)	0	.256[f]
POTS (n,%)	1 (2.3%)	2 (6.3%)	.572[f]
Hypertensive response (n,%)	3 (7%)	4 (12.5%)	.451[c]

Yrs. – years; HUTT – Head up tilt test; EVBP – Extreme variation of blood pressure; SVBP – Small variations of blood pressure; POTS- Postural orthostatic tachycardia syndrome; SD – Standard deviation; [t] – Independent Samples T test; [c] – Pearson Chi Square; [f] - Fisher's exact test

Table S2. Comparison of cardiovascular autonomic reflex test results according to history of syncope within the Bartonella IgM-seroreactive group.

	No history of Syncope N = 43	Previous Syncope N = 32	p
Abnormal HGT (n,%)	43 (100%)	32 (100%)	N/A
Abnormal OH (n,%)	9 (20.9%)	4 (12.5%)	.340[c]
Abnormal VM (n,%)	13 (30.2%)	9 (28.1%)	.843[c]
Abnormal HRB (n,%)	33 (76.7%)	19 (59.4%)	.107[c]
Abnormal HRS (n,%)	40 (93.0%)	31 (96.9%)	.632[f]

HGT-handgrip test; OH-orthostatic hypotension; VM-Valsalva maneuver; HRB-heart rate response to deep breathing; HRS-heart rate response to standing; N/A – Not Applicable; [c] – Pearson Chi Square; [f] – Fisher's exact test.

Table S3. Comparison of short-term heart rate variability according to history of syncope within the Bartonella IgM-seroreactive group.

	No history of Syncope N = 43	Previous Syncope N = 32	p
HR (bpm) (Mdn(IQR))	76 (71–85)	70 (61.5–85.5)	.346^{m}
SDNN (ms) (Mdn(IQR))	56.5 (36–70.5)	55 (46.5–85.5)	.774^{m}
RMSSD (ms) (Mdn(IQR))	29.5 (19–67)	37.5 (28–93)	.217^{m}
PNN50 (%) (Mdn(IQR))	4 (1–11.5)	8 (2–21)	.193^{m}
TP (ms^2) (Mdn(IQR))	1250.5 (736.8–1964.4)	1243.1 (492.9–2602.9)	.979m
VLF (ms^2) (Mdn(IQR))	618.9 (301.1–1322.9)	676.1 (210.7–1222.6)	.989^{m}
LF (ms^2) (Mdn(IQR))	295 (187.9–542.6)	273.1 (97.3–556.8)	.698^{m}
HF (ms^2) (Mdn(IQR))	95.8 (48.5–268.1)	161.8 (73.1–273.5)	.290^{m}
LF/HF (Mdn(IQR))	2.9 (1.75–5.9)	2.0 (1.3–2.7)	.032^{m}

HR – Heart rate; bpm – beats per minute; SDNN – Standard deviation of normal-to-normal intervals; RMSSD – Root mean square of successive differences; PNN50 – Percentage of adjacent NN intervals differing by >50 ms; TP – Total power; VLF – Very low frequency; LF – Low frequency; HF – High frequency; Mdn-median; IQR-interquartile range; ^{m} – Mann–Whitney U test.

Table S4. Comparison of long-term heart rate variability according to history of syncope within the Bartonella IgM-seroreactive group.

	No history of Syncope N = 43	Previous Syncope N = 32	p
HR (bpm) (Mdn(IQR))	80.5 (69.3 – 85.5)	71 (69.3 – 77.5)	.181[m]
SDNN (ms) (Mdn(IQR))	129 (104.5–150)	203.5 (178–210)	.008[m]
RMSSD (ms) (Mdn(IQR))	18.5 (13.5–33)	70 (54–109)	.005[m]
PNN50 (%) (Mdn(IQR))	1.5 (0–8)	25.5 (17–42)	.009[m]
TP (ms^2) (Mdn(IQR))	2017.3 (955.8–3033.4)	4497.3 (2892.7–7781.6)	.059[m]
ULF (ms^2) (Mdn(IQR))	7.9 (4.4–11.0)	37.1 (11.7–45.7)	.020[m]
VLF (ms^2) (Mdn(IQR))	1320.6 (759.5–2095.9)	2871.1 (2201.1–4834.2)	.081[m]
LF (ms^2) (Mdn(IQR))	454.5 (167.3–651.0)	1024.3 (436.6–1665.1)	.142[m]
HF (ms^2) (Mdn(IQR))	77.8 (40.8–229.5)	391.8 (157.9–1042.6)	.059[m]
LF/HF (Mdn(IQR))	3.52 (2.63–6.49)	2.35 (1.69–3.02)	.081[m]

HR – Heart rate ; bpm – beats per minute; SDNN – Standard deviation of normal-to-normal intervals; RMSSD – Root mean square of successive differences; PNN50 – Percentage of adjacent NN intervals differing by >50 ms; TP – Total power; ULF – Ultra-low frequency; VLF – Very low frequency; LF – Low frequency; HF – High frequency; m – Mann–Whitney U test.

Table S5. Comparison of ambulatory blood pressure monitoring parameters according to history of syncope within the Bartonella IgM-seroreactive group.

	No history of Syncope N = 43	Previous Syncope N = 32	p
SBP (mmHg) (Mdn(IQR))	122 (118–128)	118 (108–118)	.073m
Daytime SBP (mmHg) (Mdn(IQR))	125 (120–129.5)	118 (108–118)	.073m
Nighttime SBP (mmHg) (Mdn(IQR))	110 (110–121)	113.5 (106–117)	.534m
DBP (mmHg) (Mdn(IQR))	81 (78.5–86.5)	68.5 (68–70)	.005m
Daytime DBP (mmHg) (Mdn(IQR))	84 (80–88)	71 (71–72)	.002m
Nighttime DBP (mmHg) (Mdn(IQR))	73 (69–80.5)	67 (60–67)	.022m
SBP dipping (%) (Mdn(IQR))	6.2 (4.5–9.1)	1.35 (0.9–6.5)	.234m

SBP – Systolic blood pressure; DBP –diastolic blood pressure; Mdn – Median; IQR – Interquartile range (25%-75%); [m] – Mann–Whitney U test.

Table S6. Comparison of demographic characteristics and autonomic findings between *Bartonella henselae* IgM-reactive participants with and without myalgic encephalomyelitis/chronic fatigue syndrome (ME/CFS)

	ME/CFS absent<br>N = 52	ME/CFS present<br>N = 23	p
**Demographic characteristics**			
Age (yrs.) (mean ± SD)	46.2 ± 14.9	44.3 ± 11.1	.584[t]
Female (n,%)	34 (65.4%)	19 (82.6%)	.131[c]
**Responses during HUTT**			
Positive HUTT (n,%)	29 (55.8%)	16 (69.6%)	.261[c]
EBPV (n,%)	22 (42.3%)	8 (34.8%)	.540[c]
SBPV (n,%)	1 (1.9%)	2 (8.7%)	.221[f]
POTS (n,%)	2 (3.8%)	1 (4.3%)	1.000[f]
Hypertensive response (n,%)	5 (9.6%)	2 (8.7%)	1.000[f]

ME/CFS - myalgic encephalomyelitis/chronic fatigue syndrome; Yrs. – years; HUTT – Head up tilt test; EVBP Extreme variation of blood pressure; SVBP – Small variations of blood pressure; POTS- Postural orthostatic tachycardia syndrome; SD – Standard deviation; [t] – Independent Samples T test; [c] – Pearson Chi Square; [f] - Fisher's exact test

Table S7. Comparison of cardiovascular autonomic reflex test (CART) abnormalities between *Bartonella henselae* IgM-reactive participants with and without myalgic encephalomyelitis/chronic fatigue syndrome (ME/CFS)

	ME/CFS absent<br>N = 52	ME/CFS present<br>N = 23	p
Abnormal HGT (n,%)	52 (100%)	23 (100%)	N/A
Abnormal OH (n,%)	10 (19.2%)	3 (13.0%)	.743[f]
Abnormal VM (n,%)	15 (28.8%)	7 (30.4%)	.889[c]
Abnormal HRB (n,%)	33 (63.5%)	19 (82.6%)	.097[c]
Abnormal HRS (n,%)	49 (94.2%)	22 (95.7%)	1.000[f]

ME/CFS - myalgic encephalomyelitis/chronic fatigue syndrome; HGT-handgrip test; OH-orthostatic hypotension; VM-Valsalva maneuver; HRB-heart rate response to deep breathing; HRS-heart rate response to standing; N/A – Not Applicable; [c] – Pearson Chi Square; [f] – Fisher's exact test.

Table S8. Comparison of short term heart rate variability parameters between *Bartonella henselae* IgM-reactive participants with and without myalgic encephalomyelitis/chronic fatigue syndrome (ME/CFS)

	ME/CFS absent<br>N = 52	ME/CFS present<br>N = 23	p
Heart rate (bpm) (Mdn(IQR))	71.5 (63.5–84.5)	79.0 (72–86)	.099^{m}
HR (bpm) (Mdn(IQR))	55.5 (46.5–70.5)	47.0 (34–70)	.132^{m}
SDNN (ms) (Mdn(IQR))	39.5 (26.5–72.5)	25 (16–36)	.003^{m}
RMSSD (ms) (Mdn(IQR))	7.5 (2–18)	2 (0–4)	.005^{m}
PNN50 (%) (Mdn(IQR))	1258.2 (586.9–2258.4)	1086.2 (524.–1742.8)	.822^{m}
TP (ms^{2}) (Mdn(IQR))	598.5 (243.1–1254.4)	636.5 (310.7–1369.3)	.674^{m}
VLF (ms^{2}) (Mdn(IQR))	308.5 (198.5–556)	288.3 (132.7–396.7)	.306^{m}
LF (ms^{2}) (Mdn(IQR))	172.4 (67.8–320.5)	66.2 (46.6–137.3)	.043^{m}
HF (ms^{2}) (Mdn(IQR))	2.15 (1.40–3.25)	2.90 (2.4–6.4)	.046^{m}

ME/CFS - myalgic encephalomyelitis/chronic fatigue syndrome; HR – Heart rate; bpm – beats per minute; SDNN – Standard deviation of normal-to-normal intervals; RMSSD – Root mean square of successive differences; PNN50 – Percentage of adjacent NN intervals differing by >50 ms; TP – Total power; VLF – Very low frequency; LF – Low frequency; HF – High frequency; Mdn-median; IQR-interquartile range; ^{m} – Mann–Whitney U test.

Table S9. Comparison of 24-hour heart rate variability parameters between *Bartonella henselae* IgM-reactive participants with and without myalgic encephalomyelitis/chronic fatigue syndrome (ME/CFS)

	ME/CFS absent<br>N = 52	ME/CFS present<br>N = 23	p
HR (bpm) (Mdn(IQR))	71 (69–80)	80 (76.5–84)	.225[m]
SDNN (ms) (Mdn(IQR))	178 (134–203.5)	125 (120–143)	.291[m]
RMSSD (ms) (Mdn(IQR))	36 (16–70)	30 (25–33)	.885[m]
PNN50 (%) (Mdn(IQR))	13 (1–25.5)	7 (3.5–8)	.456[m]
TP (ms^2) (Mdn(IQR))	2927.8 (1459.2–5798.2)	2585.2 (1710.7–2751.5)	.368[m]
ULF (ms^2) (Mdn(IQR))	11.7 (6.7–37.1)	10.3 (7.2–11)	.456[m]
VLF (ms^2) (Mdn(IQR))	2381.1 (973.0–4086.7)	1563.3 (1113.4–1681.3)	.291[m]
LF (ms^2) (Mdn(IQR))	448.5 (368.4–1095.4)	711.3 (420.6–738.4)	1.000[m]
HF (ms^2) (Mdn(IQR))	157.9 (66.4–391.8)	298.8 (168.7–320.8)	1.000[m]
LF/HF (Mdn(IQR))	3.02 (2.35–5.2)	2.38 (2.31–2.87)	.555[m]

ME/CFS - myalgic encephalomyelitis/chronic fatigue syndrome; bpm – beats per minute; SDNN – Standard deviation of normal-to-normal intervals; RMSSD – Root mean square of successive differences; PNN50 – Percentage of adjacent NN intervals differing by >50 ms; TP – Total power; ULF – Ultra-low frequency; VLF – Very low frequency; LF – Low frequency; HF – High frequency; [m] – Mann–Whitney U test.

Table S10. Comparison of 24-hour ambulatory blood pressure monitoring parameters between *Bartonella henselae* IgM-reactive participants with and without myalgic encephalomyelitis/chronic fatigue syndrome (ME/CFS)

	ME/CFS absent<br>N = 52	ME/CFS present<br>N = 23	p
SBP (mmHg) (Mdn(IQR))	118 (114–122)	122 (116–125.5)	.573[m]
Daytime SBP (mmHg) (Mdn(IQR))	118 (116–125)	124 (117–127)	.692[m]
Nighttime SBP (mmHg) (Mdn(IQR))	113.5 (109–120)	110 (110–116)	.937[m]
DBP (mmHg) (Mdn(IQR))	70.5 (68–80)	81 (75–83)	.469[m]
Daytime DBP (mmHg) (Mdn(IQR))	72 (71–82)	84 (78–85)	.371[m]
Nighttime DBP (mmHg) (Mdn(IQR))	68 (67–73)	69 (66–74)	.937[m]
SBP dipping (%) (Mdn(IQR))	4.5 (0.85–6.98)	6.15 (3.08–8.72)	.937[m]

ME/CFS - myalgic encephalomyelitis/chronic fatigue syndrome; SBP – Systolic blood pressure; DBP –diastolic blood pressure; Mdn – Median; IQR – Interquartile range (25%-75%); [m] – Mann–Whitney U test.
